# Formulation of a Novel Polymeric Hydrogel Membrane for Periodontal Tissue Regeneration Using Tricalcium Phosphate-Alginate Reinforcement

**DOI:** 10.7759/cureus.57844

**Published:** 2024-04-08

**Authors:** Swetha G, Priyangha P T, Anju Cecil, Chithra S, Nidhita Suresh

**Affiliations:** 1 Department of Dentistry, Saveetha Dental College and Hospitals, Saveetha Institute of Medical and Technical Sciences, Saveetha University, Chennai, IND; 2 Department of Periodontology, Saveetha Dental College and Hospitals, Saveetha Institute of Medical and Technical Sciences, Saveetha University, Chennai, IND; 3 Department of Biomaterials, Saveetha Dental College and Hospitals, Saveetha Institute of Medical and Technical Sciences, Saveetha University, Chennai, IND

**Keywords:** hydrogel, tricalcium phosphate, polymeric membrane, hemocompatibility, alginate

## Abstract

Background: The primary goal of periodontal therapy is to facilitate the regeneration of tissues damaged by periodontal disease. In recent years, there has been a growing utilization of guided tissue regeneration (GTR) membranes with bioabsorbable properties as these membranes are increasingly employed to guide the growth of gingival tissue away from the root surface. Both resorbable and non-resorbable membranes currently employed act as physical barriers, preventing the ingrowth of connective and epithelial tissues into the defect and thereby facilitating periodontal tissue regeneration.

Objective: This study aimed to develop a polymeric hydrogel membrane reinforced with tricalcium phosphate (TCP)-alginate and assess its potential for periodontal regeneration.

Materials and methods: TCP nanoparticles were incorporated into the alginate mixture to form TCP alginate. Subsequently, the mixture was cross-linked with calcium chloride to produce a TCP-alginate polymeric hydrogel membrane. The membrane underwent hemocompatibility analysis, and also scanning electron microscopy and Fourier-transform infrared (FTIR) spectroscopy analyses were done.

Results: The SEM analysis revealed granulations and a bonded thread-like structure in the membrane, indicative of favorable conditions for cell attachment necessary for periodontal regeneration. FTIR analysis showed characteristic peaks in the spectrum, including those attributed to phosphate ion (PO4-3) at 1000.85 cm-1 and 600 cm-1, indicating the presence of β-TCP phases. Hemocompatibility assessment demonstrated a hemolysis rate of less than 5% for the TCP-alginate membrane, which is found to be within the limits.

Conclusion: The developed TCP-reinforced alginate membrane exhibited hemocompatibility and safety, suggesting its suitability for utilization in periodontal therapy as an effective regenerative material.

## Introduction

Periodontitis results in the deterioration of periodontal tissues, leading to tooth movement and eventual loss [1]. The periosteum, a thin layer of vascularized connective tissue covering the cortical bone, is vital for bone remodeling and regeneration [2]. Although conventional periodontal therapy focuses on plaque removal and inflammation reduction, it often falls short of restoring original periodontal tissues. Regenerative techniques aim to replace lost or damaged tissues to restore both form and function, but achieving complete success in humans remains challenging. Therefore, innovative regenerative strategies are crucial to assist periodontitis patients in regaining healthy periodontal tissues.

Effective barrier membranes for guided regeneration therapies must meet specific criteria, including mechanical occlusion, biological activity, biocompatibility, exposure tolerance, biodegradability, and sufficient porosity for neovascularization [3]. Additionally, membranes should be easy to handle and stable without causing tissue damage [4]. While non-resorbable expanded polytetrafluoroethylene (e-PTFE) membranes are commonly used, they require removal post-healing. Resorbable membranes address this issue but often lack adequate mechanical properties for soft tissue recovery [5].

To overcome these challenges, our study focused on developing a polymeric hydrogel membrane reinforced with tricalcium phosphate (TCP)-alginate (ALG). TCP, similar to bone phases, serves as an effective bone substitute, offering tissue compatibility, low density, stability, and wear resistance [6]. Commercially available calcium phosphates, such as hydroxyapatite and unsintered apatite, are widely used in the biomedical sector due to their beneficial properties. TCP's micromorphology, interconnecting pore structure, and resorbability align with bone remodeling, enhancing its osteoconductive function [7].

On the other hand, ALG, a naturally derived polysaccharide from seaweed, is biocompatible and non-toxic. It can be easily modified to adjust mechanical strength, degradation rate, and porosity, allowing for tailored properties based on specific application requirements [8]. ALG-based materials can be formulated as injectable hydrogels, enabling minimally invasive delivery methods for tissue engineering or wound healing applications [9]. This feature enhances patient comfort and facilitates the application of the membrane in complex anatomical sites.

Considering the individual advantages of these materials, our study aimed to fabricate a TCP-ALG-reinforced membrane and evaluate its potential for periodontal regeneration. Assessing various properties of this formulation can enhance its suitability for clinical use in periodontal therapy. 

## Materials and methods

Preparation of the ALG solution

Sodium alginate powder 0.5% (w/v) is dissolved in distilled water to produce a 3% sodium ALG solution. Derived from seaweed, sodium ALG is a biocompatible polymer that forms a thick solution when mixed with water. The mixture was continuously stirred manually at low-moderate speed, using a glass rod at a temperature of 20-25 degrees Celsius and maintained at a pH of 6.3 to ensure complete dissolution of the ALG powder, resulting in a uniform solution.

Synthesis of TCP

TCP 10% (w/v) is synthesized using the co-precipitation method. In this process, calcium and phosphate ions are combined in a solution under controlled conditions to initiate the precipitation of TCP nanoparticles. These TCP nanoparticles are then added to the previously prepared sodium ALG solution.

Preparation of the TCP-ALG polymeric hydrogel membrane

**Figure 1:**
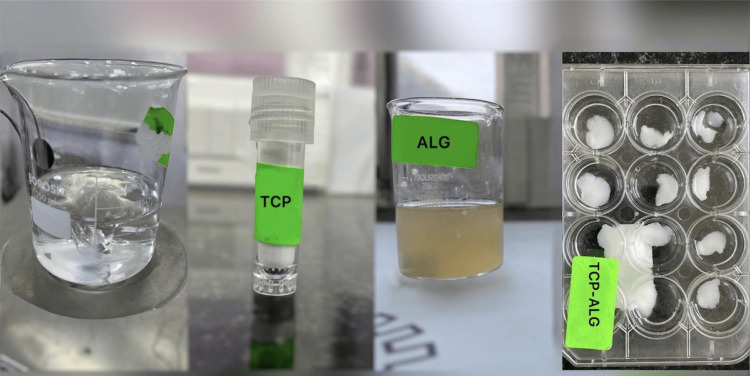
Stepwise preparation of TCP-ALG polymeric hydrogel membrane TCP-ALG: tricalcium phosphate-alginate

To crosslink the TCP-ALG mixture and form the polymeric membrane, calcium chloride (CaCl2) (0.1M) is employed as a crosslinking agent. The calcium ions present in the calcium chloride solution interact with the carboxyl groups found in the ALG polymer chains. This interaction leads to the formation of ionic crosslinks between neighboring polymer molecules. As a result, a stable and cohesive TCP-ALG polymeric hydrogel membrane is formed (Figure 1).

Characterization of the TCP-ALG polymeric membrane

Morphology of the Membrane

The membrane's microstructure analysis was performed using a scanning electron microscope (SEM), specifically the FEI Quanta FEG 650 SEM (FEI Company, Hillsboro, Oregon, United States). The SEM operated at an accelerating voltage of 2,000 kV. Images of the membrane's surface were captured at magnifications of 500x.

Fourier-Transform Infrared (FTIR) Spectroscopy Analysis

The investigation of cross-linking between ALG and TCP was conducted using a Bruker alpha II FTIR spectrometer (Bruker, Billerica, Massachusetts, United States). Attenuated total reflectance FTIR (ATR-FTIR) spectra were recorded using dry films. To create these films, hydrogels of ALG, gelatin, and TCP were poured into a polystyrene petri plate and left to dry for three days.

Hemocompatibility Assessment

The hemocompatibility assessment involved applying the solution to the membrane, and the rates of hemolysis were evaluated with both a negative and a positive control. The positive control comprised 950 μL of double-distilled water diluted with 50 μL of blood. The negative control sample consisted of 50 μL of blood and 950 μL of phosphate-buffered saline (PBS).

## Results

The membranes produced were cut into 1 × 1 cm pieces and subjected to sterilization. Following sterilization, several evaluations were conducted, including SEM analysis, FTIR analysis, and hemocompatibility assessment.

SEM analysis

**Figure 2:**
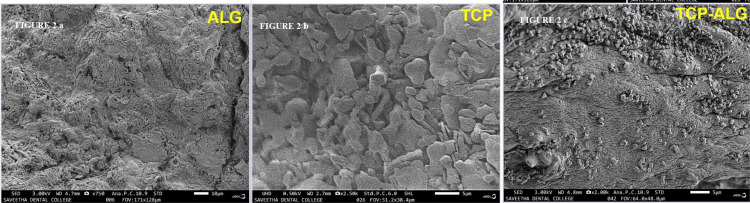
A) The morphological structure of ALG, B) the morphological structure of TCP, C) the morphological structure of the TCP-ALG membrane observed through SEM analysis TCP-ALG: tricalcium phosphate-alginate; SEM: scanning electron microscope

SEM is employed to directly observe the surface of solid objects, providing insights into the morphological structure of the membrane (Figure 2A, 2B, 2C). The SEM analysis of the TCP-ALG polymeric hydrogel membrane revealed the integration of TCP into the ALG matrix. Granulations and bonded thread-like structures were observed on the membrane surface. These granulations are presumed to be aggregates or clusters of TCP particles dispersed within the ALG matrix. The thread-like structure suggests the formation of interconnected networks or fibrils within the ALG matrix. The presence of granulations and thread-like structures on the membrane surface results in an increased surface area.

FTIR analysis

**Figure 3:**
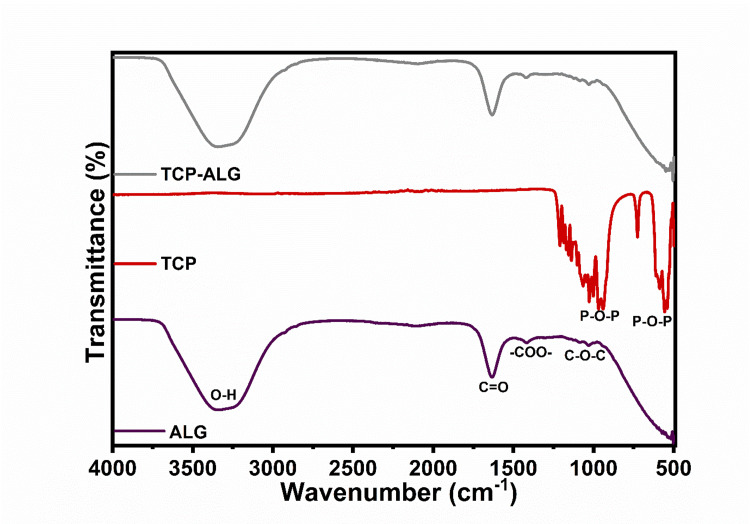
FTIR analysis with the X-axis showing wavenumber and the Y-axis showing the transmittance of ALG, TCP, and TCP-ALG membranes FTIR: Fourier transform infrared spectroscopy; ALG: alginate; TCP: tricalcium phosphate

The identification of the functional group of the synthesized calcium phosphate was carried out using FTIR analysis. As illustrated in Figure 3, phosphate bonding with oxygen was observed in these membranes. The presence of phosphate bonds in the spectrum indicates successful cross-linking between ALG and TCP. The FTIR spectrum of the samples ranged from 500 to 4000 cm^-1 after undergoing calcination at various temperatures. Peaks attributed to phosphate ions at 1000.85 cm^-1 and 600 cm^-1 indicate the presence of β-TCP phases. The calcium-to-phosphate ratio in the TCP-ALG membrane was determined to be 1.5, exhibiting an orthorhombic crystal structure. These findings suggest that the TCP-ALG membrane is highly resorbable, exhibits rapid ion release, and holds promise for positively impacting periodontal regeneration.

Hemocompatibility assay

**Figure 4:**
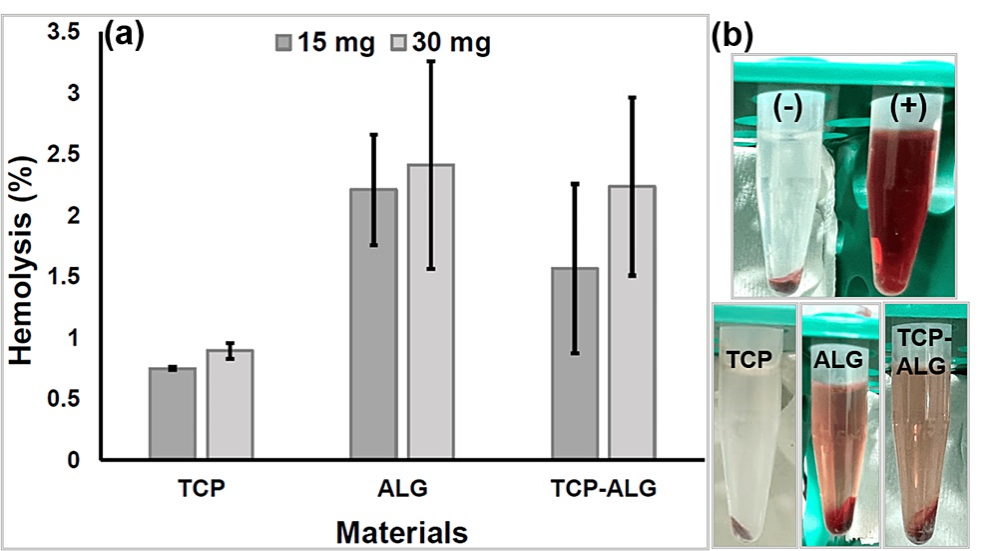
UV spectrophotometer analysis of the hemolysis of the TCP, ALG, and TCP-ALG membranes at 15mg and 30 mg UV: ultraviolet; TCP: tricalcium phosphate; ALG: alginate

The study assessed the hemocompatibility of membranes made of TCP only, ALG only, and a combination of TCP-ALG (Figure 4). Hemolysis, which refers to the rupture or lysis of RBCs, is a critical parameter for evaluating the compatibility of materials with blood. Hemolysis was quantified using an ultraviolet (UV)-visible spectrophotometer, where the absorbance of the hemoglobin released from lysed RBCs in a solution was measured. Higher absorbance indicates greater hemolysis, as more hemoglobin is released into the solution.

To validate the hemolysis assay, both positive and negative controls were utilized. In the control samples, all RBCs were ruptured, resulting in complete hemolysis and a red coloration of the solution due to the release of hemoglobin.

The study outcomes indicated that the TCP-ALG membrane had a hemolysis rate of less than 5%. The specific values provided (1.5% for 15 mg and 2.3% for 30 mg) represent the percentage of hemolysis observed at different concentrations of the TCP-ALG membrane. Overall, the study demonstrated that the TCP-ALG membrane exhibits low hemolytic activity, making it suitable for biomedical applications requiring contact with blood.

## Discussion

Periodontal regeneration aims to restore damaged tissue surrounding teeth affected by periodontal disease. To enhance biological activity during regeneration, scaffolds, cells, and growth factors are utilized. Since the introduction of the guided tissue regeneration (GTR) concept, various membranes have been employed. The identification and isolation of cells capable of rebuilding the periodontal ligament, cementum, and connective tissue are crucial. Eliminating epithelial cells from the periodontal defect is a primary objective, as they have a higher turnover rate than osteoblasts and fibroblasts, which are the primary targets for treatment [10].

GTR methods have successfully treated periodontal issues and stimulated bone regrowth. GTR creates a gap around defects using membranes as mechanical barriers, preventing surrounding connective tissues from competing for space and facilitating new bone formation. Membranes used in GTR therapy must be biocompatible, degrade appropriately, and possess physical and mechanical stability. For cellular adaptability and nutritional support, GTR membranes should also be permeable [11].

Numerous studies have highlighted the individual advantages of ALG and TCP as membranes for periodontal regeneration. This study combines these materials to formulate a polymeric hydrogel membrane, leveraging their synergistic effects. TCP, chosen for its biocompatibility and reabsorption properties, is a scaffold for bone formation as it degrades. ALG, relatively inexpensive and readily available, offers a cost-effective option for tissue regeneration compared to synthetic alternatives. Its property of forming a hydrogel in the presence of divalent cations such as calcium ions facilitates the encapsulation and delivery of cells, growth factors, or drugs within the membrane, which are essential for tissue regeneration [12].

In a study focusing on 3D-printed hydroxyapatite and tricalcium phosphates-based scaffolds for alveolar bone regeneration in animal models, promising outcomes were observed, showcasing new bone formation without any inflammatory reactions across different animal species [13]. Another investigation delved into the synthesis and properties of β-TCP for bone substitution. The study concluded that β-TCP exhibits synthetic, osteoconductive, and osteoinductive properties and undergoes cell-mediated resorption, making it a potent bone graft substitute [14]. Furthermore, a review of the current application of β-TCP in bone repair emphasized the advantages of β-TCP, for repairing and regenerating damaged bone tissues. The inclusion of additional materials to adjust its mechanical characteristics and degradation rate was recommended to enhance its efficacy. In comparison to alternative biological materials, β-TCP was identified as having a more suitable degradation rate, which is advantageous for delivering therapeutic dissolution products [15].

Incorporating various materials into β-TCP to modulate its mechanical performance has been highlighted in several studies, reinforcing its importance. With this insight, our study combined the properties of ALG and TCP to develop a hydrogel polymeric membrane promising enhanced therapeutic efficacy and offering a multifaceted approach to treating periodontal diseases.

Furthermore, studies on the fabrication and properties of ALG-hydroxyapatite biocomposites and the synthesis and characterization of a hydroxyapatite-sodium ALG-chitosan scaffold for bone regeneration demonstrated promising results [16,17]. These studies concluded that the fabricated scaffolds exhibited high pore volume with adequate size and interconnectivity for osseous tissue regeneration and possessed good physical, chemical, antibacterial, and osteogenic properties.

In our study, the formulated polymeric hydrogel membrane containing ALG and TCP underwent in-vitro characterization. The SEM analysis revealed the incorporation of TCP into the ALG membrane, displaying granulations and a bonded thread-like structure. The granular formations seen in the SEM images can enhance the surface roughness and porosity of the membrane, while the interconnected thread-like structure can bolster its mechanical strength and stability, promoting cell adhesion and proliferation. This increased surface area holds promise for diverse applications like tissue engineering, drug delivery, and membrane filtration, providing more sites for interactions with the surrounding environment, such as cell attachment or molecule adsorption. These findings are consistent with previous research that investigated the synthesis and characterization of antibacterial drug-loaded β-TCP powders for bone engineering applications, revealing agglomerated acicular prismatic particles with a beaded appearance [18].

In FTIR analysis, the peaks observed at 1000.85 cm⁻¹ and 600 cm⁻¹ are indicative of phosphate groups, specifically attributed to phosphate ions. The peak at 1000.85 cm⁻¹ corresponds to stretching vibrations, while the one at 600 cm⁻¹ relates to bending vibrations of these phosphate groups. The presence of these peaks in the FTIR spectrum suggests the existence of β-TCP phases in the analyzed sample, as β-TCP is known for exhibiting distinctive phosphate group vibrations within this spectral range. This conclusion is reinforced by a study that also identified similar peaks at 1030.85 cm⁻¹ and 573.19 cm⁻¹ [19].

The hemolysis test showed a biocompatible hemolysis rate of less than 5% for the TCP-ALG membrane, which is considered biocompatible according to the American Standard for Testing Materials. The incorporation of TCP into the ALG membrane may have contributed to its hemocompatibility, possibly through surface modifications or interactions that reduce RBC lysis, thus supporting its potential use in medical devices without adverse effects on blood cells.

Periodontal treatment aims to resolve inflammation and achieve tissue regeneration effectively [20]. For a barrier membrane to be effective, it must fulfill essential design criteria, one of which is biocompatibility, to prevent an immune response and facilitate practical application [21].

Based on our study, the TCP-ALG polymeric hydrogel membrane shows promise for periodontal regenerative therapy. Comparative studies with other membranes would provide a comprehensive understanding of their effectiveness and clinical benefits in real-world scenarios.

While these membranes show promise for regeneration in vitro, their clinical application has yet to be established. Therefore, further in-vivo clinical studies are necessary to evaluate the clinical effectiveness of the formulated polymeric hydrogel membrane as a regenerative material in periodontal therapy.

## Conclusions

The findings from this investigation suggest that the developed polymeric hydrogel membrane incorporating TCP-ALG is hemocompatible and versatile rendering it suitable for application in periodontal therapy as an effective regenerative material. Nonetheless, additional research is required to comprehensively analyze and comprehend the impact of the formulated hydrogel on clinical periodontal parameters. Enhanced comprehension of the factors that impact the regenerative process is expected to improve the predictability of therapy for bone defects around natural teeth and implants.
